# Incidence and Risk Factors of Peri‐Implantitis Over Time—A Prospective Cohort Study

**DOI:** 10.1111/jre.13367

**Published:** 2025-01-13

**Authors:** Mario Romandini, Cristina Lima, Diogo Banaco, Rita Azevedo, Mariano Sanz

**Affiliations:** ^1^ Section of Periodontology, Faculty of Odontology University Complutense Madrid Spain

**Keywords:** dental implants, dental prosthesis, epidemiologic factors, epidemiology, maintenance, peri‐implant diseases, periodontal diseases

## Abstract

**Aim:**

This prospective cohort study aimed to evaluate the incidence and risk/protective factors of peri‐implantitis over time.

**Methods:**

A university‐representative cohort was evaluated at baseline and after a mean follow‐up time of 3.9 years. The main outcome was the incidence of peri‐implantitis, defined as bone loss > 1 mm between the two examinations in implants showing bleeding on probing. Putative risk/protective factors assessed at baseline were tested through multilevel (mixed‐effects) logistic regression analyses.

**Results:**

A total of 73 patients with 322 implants were included. During the follow‐up period, 14 implants (4.3%) were lost in 9 patients (12.3%). Incidence of peri‐implantitis was observed in 22.2% of patients and 9.4% of implants. In the final multilevel multiple logistic regression model, the following factors were associated with occurrence of peri‐implantitis: periodontitis severity (stage IV periodontitis: OR = 41.29; 95% CI: 4.10–415.54), periodontal bone loss/age ratio (> 1: OR = 8.87; 95% CI: 1.47–53.73), smoking (current smokers: OR = 7.84; 95% CI: 1.83–33.50), sleep duration (> 7 h: OR = 19.97; 95% CI: 1.69–236.39), implant location (incisor: OR = 60.60; 95% CI: 4.04–908.33), restoration type (full‐arch fixed restorations: OR = 89.84; 95% CI: 3.66–2202.97), and restoration margin location (juxta‐marginal: OR = 14.17; 95% CI: 1.20–166.76). Keratinized tissue width assessed at baseline was not associated with incidence of peri‐implantitis.

**Conclusion:**

Approximately one in five patients and one in 10 implants experienced incident peri‐implantitis over a nearly four‐year period. Periodontitis (Stage IV and Grade C), lifestyles (smoking and sleep duration), implant location, and prosthetic factors (restoration type and margin location) emerged as risk factors for peri‐implantitis.

## Graphical abstract

Identifying true risk factors requires a longitudinal evaluation to verify the temporality criterion. This study identified periodontitis (stage and grade), lifestyle behaviours (smoking and sleep duration), implant location, and prosthetic factors (restoration type and margin location) as risk factors for peri‐implantitis.

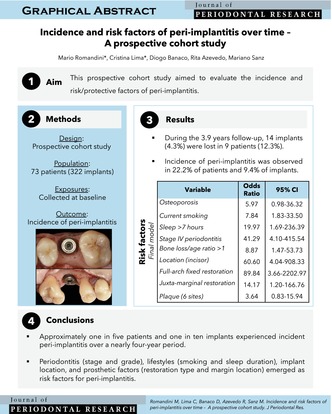

**Summary**

- Background
  ○ Identifying true risk and protective factors requires a longitudinal evaluation to verify the temporality criterion.
  ○ Since many identified putative risk factors for peri‐implantitis are either not modifiable or not amenable to randomized clinical trials due to ethical reasons, cohort studies are the most appropriate design.
  ○ However, only a few cohort studies exist in the field, mostly at risk of selection, confounding, or information bias.
- Added value of this study
  ○ Approximately one in five patients and one in 10 implants experienced incident peri‐implantitis over a nearly four‐year period.
  ○ This study identified periodontitis (stage and grade), lifestyle behaviours (smoking and sleep duration), implant location, and prosthetic factors (restoration type and margin location) as risk factors for peri‐implantitis within a longitudinal framework of a representative sample.
- Clinical implications
  ○ Clinicians should address these factors when they are modifiable and consider them for implementing more stringent preventive measures when they are not modifiable.

## 1 Introduction

Given its high prevalence [1, 2, 3, 4, 5], rapid progression [6], and the limited efficacy of current treatment procedures [7, 8, 9, 10, 11], primary prevention and early diagnosis [12, 13] are essential in managing peri‐implantitis. The main strategies of peri‐implantitis prevention, rely on (i) treating peri‐implant mucositis, since it is considered its precursor [14, 15] and (ii) implementing interventions aimed at controlling the modifiable risk factors [1, 16, 17, 18, 19, 20]. A recent cross‐sectional study performed by our research group identified several risk indicators for peri‐implantitis, including moderate/severe periodontitis, smoking, implant brand and malposition, restorative factors, and plaque [2]. These risk indicators were consistent with other studies with similar designs [1, 5, 21, 22]. However, the identification of true risk/protective factors requires a longitudinal evaluation to verify the temporality criterion, ideally through randomized clinical trials to minimize the risk of confounding bias [23]. Since many of the identified putative risk factors for peri‐implantitis are either not modifiable or not amenable to randomized clinical trials due to ethical reasons, cohort studies are the most appropriate design. However, only a few cohort studies exist in the field [24, 25, 26], mostly at risk of selection, confounding, or information bias. Therefore, this prospective cohort study aimed to evaluate the incidence of peri‐implantitis over time and associated risk/protective factors.

## 2 Methods

This manuscript adheres to the STrengthening the Reporting of OBservational studies in Epidemiology (STROBE) guidelines [27, 28]. The study was conducted in accordance with the Declaration of Helsinki for human studies, and its research protocol was ethically approved (19/182‐E; 22/385‐EC_P) by the CEIm Hospital Clínico San Carlos, Madrid, Spain.

### 2.1 Population

**FIGURE 1:**
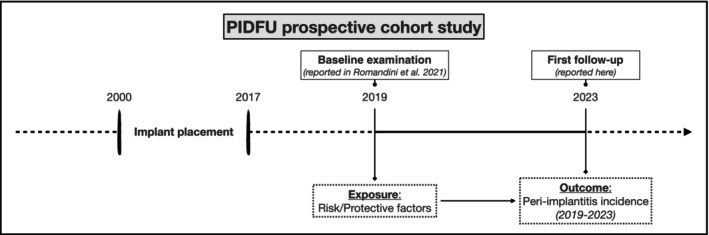
PIDFU prospective cohort study design.

The Peri‐Implant Diseases Follow‐Up (PIDFU) study is an ongoing prospective cohort study based on the repeated follow‐ups over time of a previously reported university‐representative population [2, 29, 30]. During 2019, 99 patients with 458 implants, identified through a stratified multistage sampling process among patients who received dental implants from 2000 to 2017 in the Department of Periodontology at Complutense University of Madrid, were initially examined both clinically and radiographically. This examination served as the baseline for this prospective cohort study (Figure 1). In 2023, the same patients were invited to participate to the first follow‐up examination through a minimum of five different telephonic attempts made on different days.

### 2.2 Risk/Protective Factors Tested (Exposure)

The full list of patient‐ and implant‐level variables tested as putative risk/protective factors is reported in Table 1. They were collected during the baseline examination, and their assessment methods are detailed in the baseline study publication [2].

**TABLE 1:** Putative risk/protective factors tested.

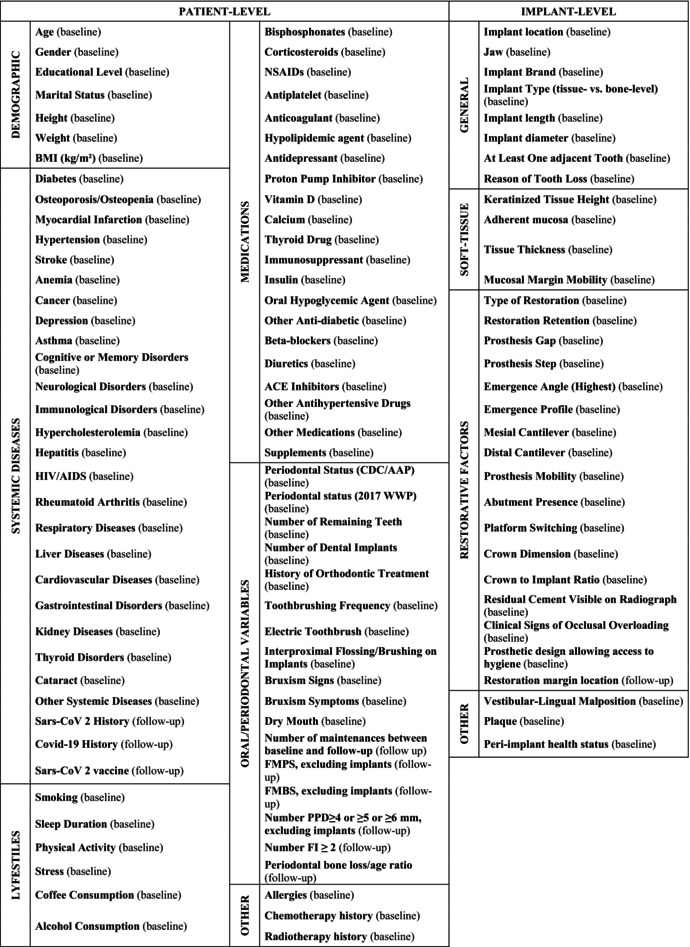

During the presently reported follow‐up examination, patients additionally self‐reported (no, yes) about their SARS‐CoV‐2 and COVID‐19 history and vaccination status. Additionally, patients indicated the number of supportive peri‐implant care (SPIC) recalls and any treatments (non‐surgical and/or surgical) performed on the study implants since the baseline examination. For interventions conducted in‐house, information regarding the number of SPIC recalls and treatments was cross‐verified by accessing patient files. Regular maintenance was defined as participating in an average of ≥ 1 SPIC recalls per year.

A full‐mouth periodontal examination was also performed on the residual dentition, and full‐mouth plaque and bleeding scores (FMPS/FMBS), the number of probing pocket depth (PPD) exceeding different thresholds (≥ 4, ≥ 5, ≥ 6 mm), and the number of furcation involvements [31] (FI) ≥ 2 were recorded. At implant‐level, the location of the restoration margin in relation to the soft‐tissue margin (sub‐, juxta‐, or supra‐marginal) was recorded based on its most apical position around the implant. An orthopantomography was also performed, and the periodontal bone loss/age ratio was measured in the most severely affected tooth [32, 33].

### 2.3 Peri‐Implantitis Onset/Progression (Outcome)

The primary outcome of the study was peri‐implantitis onset/progression, defined as the incidence of bone loss > 1 mm between the baseline and follow‐up examinations in implants showing bleeding on probing (BoP) at one or more sites during follow‐up. The 1 mm threshold was chosen to minimize the risk of misclassification bias due to measurement error. Additional bone loss thresholds (> 0.5 mm and > 2 mm) were also considered for descriptive purposes.

At the follow‐up examination, BoP was recorded at six sites per implant by a calibrated clinical examiner (CL). New standardized peri‐apical radiographs of the included implants were obtained from the Radiology Department using the parallel technique. The marginal bone level at the follow‐up examination was assessed by the same calibrated radiographic examiner from the baseline study (CL), applying the same measurement protocol described in the original publication [2]. Briefly, the radiographic bone level was measured at the mesial and distal aspects of each implant as the distance in millimeters between the intra‐osseous portion of the implant (excluding any polished collar) and the first clearly visible contact between the implant surface and the bone. A software program (Autocad 2016 TM, AutoDesk Inc. San Rafael, CA, USA) was used, and the inter‐thread pitch distance reported by the manufacturer or the length of the implant was considered for calibration. The highest value between the mesial and distal measurements was recorded as the bone level for that implant. Bone loss was calculated as the difference in bone levels between the two examinations. The radiographic examiner previously demonstrated an excellent intra‐rater agreement after re‐measuring 50 randomly selected radiographs (ICC = 0.98; 95% CI: 0.96–0.99; *p* < 0.001).

### 2.4 Data Analysis

Statistical analyses were performed with STATA SE version 18.0 software (StataCorp LP). The characteristics of the study population/implants were summarized. Incidence of peri‐implantitis onset/progression was described at both patient‐ and at implant‐level.

Risk/protective factors for peri‐implantitis onset/progression were studied using multilevel (mixed‐effects) logistic regression analyses, accounting for the clustering of multiple implants within the same patients. Each putative factor was tested individually by adding it to an empty model with the peri‐implantitis onset/progression as the dependent variable and testing its significance. All variables with a *p* < 0.10 were included in an intermediate multiple regression model, and non‐significant variables were sequentially removed. The final model integrated all factors that remained with a *p* < 0.10. However, for results interpretation, statistical significance was a priori set at *p* < 0.05. Sensitivity analyses were performed by adjusting the final model a priori for the mean frequency of maintenance per year, treatments performed during follow‐up, and peri‐implant health status at baseline.

## 3 Results

From the baseline sample of 99 patients, 14 declined to participate in the follow‐up visit, three moved to a different city, two missed the scheduled appointment, one passed away, and six could not be reached despite multiple telephone attempts. Consequently, 73 patients with 322 implants were clinically evaluated after a mean follow‐up time of 3.9 years (SD = 0.3; min: 3.0; max: 4.6). The general characteristics of the population and implants examined at follow‐up are detailed in Tables 2 and 3, and they are consistent with those of the entire baseline population. Most of the included patients were women (61.6%), had a mean age of 62.8 years, had a diagnosis of stage III‐IV periodontitis (57.5%), and were currently nonsmoking (80.8%) at baseline. Most of the study implants were located in the maxilla (55.6%), and were part of implant‐supported bridge restorations (58.4%). At baseline, 91 implants (28.2%) in 38 patients (52.1%) were diagnosed with peri‐implantitis.

**TABLE 2:** General characteristics of the study population.

	Baseline population *(N = 99)*	Follow‐up population *(N = 73)*
Age (baseline) (years), mean (SD)	63.7 (9.3)	62.8 (8.0)
Gender, *N* (%)		
Male	39 (39.4)	28 (38.4)
Female	60 (60.6)	45 (61.6)
BMI (baseline) (kg/m^2^), mean (SD)	25.6 (3.7)	25.7 (3.8)
Diabetes status (baseline), *N* (%)		
No diabetes	83 (83.8)	61 (83.6)
Diabetes	16 (16.2)	12 (16.4)
Smoking (baseline), *N* (%)		
Non‐smokers	41 (41.4)	28 (38.3)
Former smokers	40 (40.4)	31 (42.5)
Current smokers	18 (18.2)	14 (19.2)
Periodontitis severity (2017 WWP) (baseline), *N* (%)		
No Periodontitis	7 (7.2)	4 (5.5)
Stage 1	11 (11.3)	7 (9.6)
Stage 2	19 (19.6)	14 (19.2)
Stage 3	30 (30.9)	25 (34.2)
Stage 4	21 (21.7)	17 (23.3)
Edentulous	9 (9.3)	6 (8.2)
Peri‐implant status (baseline), *N* (%)		
Peri‐implant health	1 (1.0)	0 (0.0)
Peri‐implant mucositis	11 (11.1)	10 (13.7)
Pre‐peri‐implantitis	31 (31.3)	25 (34.2)
Peri‐implantitis	56 (56.6)	38 (52.1)
Maintenance compliance (during follow‐up), *N* (%)		
Regular maintenance	NA	9 (12.3)
Not regular maintenance	NA	64 (87.7)

*Note:* Total number varies according to missing data for each variable. Regular maintenance was defined as participating in an average of ≥ 1 supportive peri‐implant care recalls per year.

Abbreviations: *N*, number; NA, not applicable; SD, standard deviation.

**TABLE 3:** General characteristics of the study implants.

	Whole baseline implants *(N = 458)*	Implants analyzed at follow‐up *(N = 322)*
Jaw, *N* (%)		
Maxilla	253 (55.2)	179 (55.6)
Mandible	205 (44.8)	143 (44.4)
Position, *N* (%)		
Anterior (canine‐canine)	83 (18.1)	54 (16.8)
Posterior	375 (81.9)	268 (83.2)
Implant brand, *N* (%)		
S	230 (50.7)	173 (53.9)
N	57 (12.6)	38 (11.8)
A	76 (16.7)	45 (14.0)
Other	91 (20.0)	65 (20.3)
Implant Length (mm), mean (SD)	9.9 (1.7)	9.84 (1.71)
Implant Diameter (mm), mean (SD)	4.1 (0.4)	4.1 (0.4)
Type of prosthesis (baseline), *N* (%)		
Single crown	136 (29.7)	103 (32.0)
Bridge	267 (58.3)	188 (58.4)
Overdenture	14 (3.1)	8 (2.5)
Full‐arch fixed restoration	41 (8.9)	23 (7.1)
Prosthesis retention (baseline), *N* (%)		
Cemented	218 (47.6)	163 (50.6)
Screw‐retained	226 (49.3)	151 (46.9)
Locator	8 (1.8)	2 (0.6)
Bar	6 (1.3)	6 (1.9)
Peri‐implant status (baseline), *N* (%)		
Peri‐implant health	39 (8.5)	25 (7.8)
Peri‐implant mucositis	146 (31.9)	107 (33.2)
Pre‐peri‐implantitis	145 (31.7)	99 (30.8)
Peri‐implantitis	128 (27.9)	91 (28.2)

*Note:* Total number varies according to missing data for each variable. Implant brands: S, Straumann; N, Nobel Biocare; A, AstraTech.

Abbreviations: *N*, number; SD, standard deviation.

### 3.1 Interventions During Follow‐Up

Most of the included implants (189/58.7%) did not receive any treatment during the follow‐up period. Non‐surgical treatment only was performed in 79 (24.6%) implants, whereas 18 (5.6%) underwent surgical treatment. Finally, 14 implants (4.3%) underwent removal, whereas no reliable information on previous interventions was available for the remaining 22 implants (6.8%).

The mean number of SPIC visits during follow up was 2.0 (SD = 1.5; min: 0; max: 7), with an average of 0.5 per year (SD = 0.4; min: 0; max: 1.9). 12.3% of patients were under regular maintenance, since they had an average of more than one SPIC visit per year.

### 3.2 Incidence of Peri‐Implantitis Onset/Progression

In addition to the 14 implants lost (9 patients), 10 implants had missing/unreadable follow‐up radiographs. Therefore, peri‐implantitis onset/progression was assessed on 298 implants.

An incidence of bone loss > 1 mm during follow‐up was observed in 16 out of 72 patients (22.2%) and 28 out of 298 implants (9.4%) evaluated radiographically (Table 4). Bone loss > 1 mm was always associated with the presence of BoP at follow‐up. Sixteen events (5.4%) corresponded to new peri‐implantitis cases (i.e., peri‐implantitis onset), whereas 12 (4.0%) involved further bone loss of implants already diagnosed with peri‐implantitis at baseline (i.e., peri‐implantitis progression). Peri‐implantitis onset was mostly observed among implants diagnosed with peri‐implant mucositis (9%–8.6%) or pre‐periimplantitis (6%–6.3%) at baseline, compared with only one implant with peri‐implant health (4.8%) at baseline (Table 5). Most peri‐implantitis progressions were observed around implants that underwent surgical treatment during follow‐up (40.0%), which might have been performed because of the incident bone loss. Sixty‐five (84.4%) implants diagnosed with peri‐implantitis at baseline did not show further bone loss.

**TABLE 4:** Incidence of bone loss during follow‐up according to different peri‐implant diagnosis at baseline.

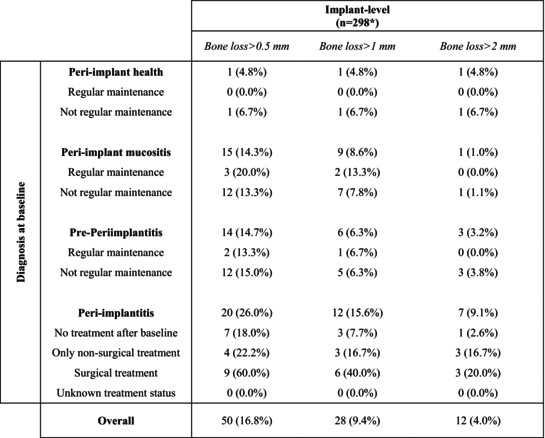

*Note:* Regular maintenance was defined as participating in an average of ≥ 1 supportive peri‐implant care recalls per year.

* Missing data: 24 implants (10 had missing/unreadable radiographs; 14 were lost/removed during follow‐up).

**TABLE 5:** Occurrence of peri‐implant diseases and implant loss during follow‐up according to different peri‐implant diagnosis at baseline.

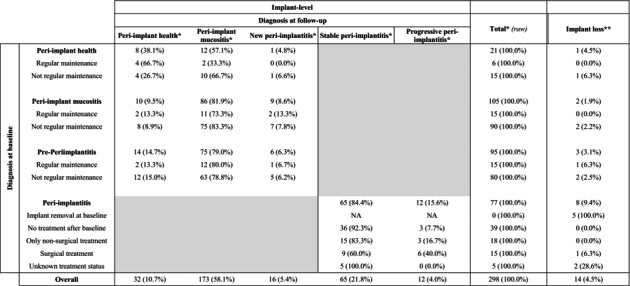

*Note:* Regular maintenance was defined as participating in an average of ≥ 1 supportive peri‐implant care recalls per year.

Abbreviation: NA, not applicable.

* Percentages refer to implants evaluated radiographically (*n* = 298) (missing data: 10 implants due missing/unreadable radiographs, 14 implants lost during follow‐up).

** Percentages refer to implants evaluated clinically (i.e., including implants lost during follow‐up) (*n* = 312) (missing data: 10 implants due missing/unreadable radiographs).

### 3.3 Risk/Protective Factors Associated With Peri‐Implantitis Onset/Progression

The distribution of the tested risk/protective factors according to peri‐implantitis incidence is detailed in Tables S1 and S2.

In the multi‐level simple regression analyses, the following patient‐level exposure variables were associated with peri‐implantitis (*p* < 0.10): marital status, height, osteoporosis/osteopenia, myocardial infarction, hepatitis, smoking, sleep duration, alcohol consumption, intake of bisphosphonates, vitamin D, or immunosuppressants, periodontitis severity (2017 WWP), number of remaining teeth, electric toothbrush use, and periodontal bone loss/age ratio (Table A1). The following implant‐level variables were also associated with peri‐implantitis: implant location, presence of at least one adjacent tooth, type of restoration, prosthetic design, restoration margin location, implant malposition, and plaque (Table A2).

In the final multilevel multiple logistic regression model, the following factors remained significant at the *p* < 0.05 level: periodontitis severity (stage IV periodontitis: OR = 41.29), periodontal bone loss/age ratio (> 1: OR = 8.87), smoking (current smokers: OR = 7.84), sleep duration (> 7 h: OR = 19.97), implant location (incisor: OR = 60.60), restoration type (full‐arch fixed restorations: OR = 89.84), and restoration margin location (juxta‐marginal: OR = 14.17) (Table 6). Osteoporosis/osteopenia (yes: OR = 5.97) and plaque (6 sites: OR = 3.64) also entered the final model (*p* < 0.10), despite being not statistically significant (*p* > 0.05). Sensitivity analyses, adjusting the final model a priori for frequency of maintenance per year, treatments performed during follow‐up, and peri‐implant health status at baseline, showed results consistent with the main analyses.

**TABLE 6:** Risk/protective indicators associated with incidence of peri‐implantitis during follow‐up: multilevel multiple logistic regression analysis.

Variable	Empty model		Final model		
	OR	95% CI	OR	95% CI	*p*
Fixed part					
Intercept	0.04	0.01–0.12	0.00	0.00–0.01	
Osteoporosis/osteopenia (yes) (baseline)			5.97	0.98–36.32	0.052
Smoking (baseline)					
Non‐smoker			Ref	Ref	Ref
Current smoker			7.84	1.83–33.50	**0.005**
Sleep duration (baseline)					
< 7 h			0.80	0.20–3.21	0.751
7–8 h			Ref	Ref	Ref
> 7 h			19.97	1.69–236.39	**0.018**
Periodontitis severity (2017 WWP) (baseline)					
No periodontitis or SI‐III periodontitis			Ref	Ref	Ref
Stage 4 periodontitis			41.29	4.10–415.54	**0.002**
Edentulous			NE	NE	NE
Periodontal bone loss/age ratio (follow‐up)					
≤ 1			Ref	Ref	Ref
> 1			8.87	1.47–53.73	**0.017**
Implant location (baseline)					
Molar			Ref	Ref	Ref
Incisor			60.60	4.04–908.33	**0.003**
Canine			NE	NE	NE
Premolar			0.90	0.28–2.93	0.862
Restoration type (baseline)					
Single crown			Ref	Ref	Ref
Bridge			1.19	0.27–5.30	0.817
Overdenture			NE	NE	NE
Full‐arch fixed restoration			89.84	3.66–2202.97	**0.006**
Restoration margin location (follow‐up)					
Supra‐marginal			Ref	Ref	Ref
Sub‐marginal			5.97	0.54–66.02	0.145
Juxta‐marginal			14.17	1.20–166.76	**0.035**
Plaque (baseline)					
0–5 sites			Ref	Ref	Ref
6 sites			3.64	0.83–15.94	0.086
Random part					
Patient variance	2.60	0.79–8.53	0.00	0.00–0.00	
AIC	177.61		119.64		

*Note:* *p* < 0.05 are reported in bold.

Abbreviations: AIC, Akaike‘s information criterion; CI, confidence interval; OR, odds ratio; Ref, reference category.

## 4 Discussion

In this prospective cohort study, the incidence of peri‐implantitis was ~10% over a period of ~4 years, comprising 5.4% new cases and 4.0% progressions. Identified risk factors for peri‐implantitis included Stage IV periodontitis, a periodontal bone loss/age ratio > 1, current smoking, sleep duration > 7 h, incisor position, full‐arch fixed restorations, and juxta‐marginal margin location. Additionally, there was a non‐statistically significant trend for plaque and osteoporosis. Analyses adjusted for the frequency of maintenance per year, treatments performed during follow‐up, and peri‐implant health status at baseline showed results consistent with the main analyses.

Few prospective cohort studies are available for comparison, with most existing evidence at risk of selection, confounding, or information bias. Costa el al. [14] followed 80 patients for 5 years, reporting a higher incidence of peri‐implantitis (31.2%). Differences in follow‐up length and case definitions may explain the discrepancies in peri‐implantitis incidence over time.

The history of periodontitis has been consistently linked to peri‐implantitis [34]; however, only a few studies have a longitudinal design [25, 35], and none used the current classification system [32]. Consistent with the baseline study [2], our findings indicate that the most severe forms of periodontitis are the ones at higher risk of peri‐implantitis. According to the 2017 classification, Stage IV cases are the ones where dental implants are used to restore masticatory function after periodontitis‐related tooth loss. Furthermore, the periodontal bone loss/age ratio represents the indirect criterion for defining the rate of progression of periodontitis (grade) in cases lacking previous records. A periodontal bone loss/age ratio > 1 (Grade C) emerged as a risk factor for peri‐implantitis, highlighting how the most aggressive forms of periodontitis are also at higher risk, as previously reported for the 1999 classification system [36].

Although some cohort studies have indicated smoking as a risk factor for peri‐implantitis [24], the majority have not [37, 38, 39]. This controversial relationship may be due to a masking effect by periodontitis [1]. Consistent with the baseline study [2], current smokers exhibited a higher risk of peri‐implantitis in the present study, possibly caused by its microvascular effects and the associated changes in peri‐implant microbiota [40].

The observed higher risk of peri‐implantitis incidence in long sleepers underlines the relevance of healthy lifestyles beyond non‐smoking for promoting peri‐implant health. Despite being novel for peri‐implantitis, this finding is consistent with periodontal literature [41, 42, 43, 44]. Longer sleep durations may additionally indicate poor general or mental health status, specifically depression and related medication intakes, which have been previously strongly related to peri‐implantitis and implant loss [45, 46].

Several implant‐level variables were also associated with the incidence of peri‐implantitis. The higher risk observed in anterior zones aligns with previous cross‐sectional studies [5] and recent reports indicating elevated rates of implant loss in the anterior mandible [47]. The unique anatomical and histological characteristics of these zones, combined with the specific surgical and prosthetic protocols aimed at maximizing esthetic outcomes ‐ yet potentially limiting access for biofilm removal ‐ may contribute to this increased risk. Similarly, the higher risk of peri‐implantitis observed for full‐arch restorations, apart from being interpretable as a proxy of the most severe forms of periodontitis, may also be related to the increased difficulty in accessing and performing self‐administred oral hygiene procedures [5, 48]. The formation of sub‐marginal‐ microbiological niches may also explain the association between restoration margin location and peri‐implantitis, highlighting the relevance of restoration designs in mantaining peri‐implant health [1, 49].

Although there is substantial evidence supporting the role of biofilm accumulation hosting a dysbiotic microbiota as a risk factor for peri‐implantitis [2, 17, 50, 51, 52], the presence of plaque was not longitudinally associated with incident peri‐implantitis in this cohort, despite entering in the final model. This finding may be explained by potential variation in plaque control over time. In addition to plaque, also implant brand and malposition, keratinized tissue width, and maintenance frequency were not associated with peri‐implantitis. The first three factors may have lacked statistical power, as suggested by their non‐significant tendencies for association in simple regression analyses. In contrast, maintenance frequency could have been subject to a masking effect from periodontitis, because the most severe forms of periodontitis may necessitate more frequent maintenance recalls.

The relevance of the present findings lies in the prospective cohort study design. The risk of selection bias is mitigated by the representative methods used to select the study population at baseline and the similar characteristics of patients examined at follow‐up. The use of multi‐level regression analyses, adjusted for other risk factors, controls the risk of confounding bias. Additionally, the use of direct evidence to assess bone loss within a longitudinal framework and the consistent assessment performed by the same calibrated examiner from the baseline study help minimize the risk of information bias. However, some limitations need consideration. The absence of previous studies with a similar design and scope necessitated an exploratory approach for this study. Consequently, while limited statistical power may have prevented the identification of relevant additional risk factors, the risk of a type I error cannot be ruled out. Moreover, some risk indicators were self‐reported, whereas others were assessed using methods that do not meet gold‐standard criteria (e.g., mucosal thickness measured with a probe), introducing a potential risk of information bias for these exposures. Additionally, two of the emerged risk factors (periodontal bone loss/age ratio and restoration margin location) were only assessed at the follow‐up examination, preventing verification of their presence before peri‐implantitis occurred. Finally, despite selected through a stratified multistage sampling process, the analyzed population is derived from a single center primarily including periodontitis patients in a specific country. Therefore, the generalizability of these findings to different settings needs to be verified by future studies.

## 5 Conclusions

In this prospective cohort study, approximately one in five patients and one in ten implants experienced incident peri‐implantitis over a nearly four‐year period. Periodontitis (stage and grade), lifestyle behaviours (smoking and sleep duration), implant location, and prosthetic factors (restoration type and margin location) were identified as risk factors for peri‐implantitis. Clinicians should address these factors when modifiable or consider them for implementing more stringent preventive measures when not modifiable.

## Author Contributions

M.R. contributed to study conception and design, to data acquisition, analysis and interpretation, and manuscript drafting. C.L. contributed to data acquisition, analysis and interpretation, and manuscript drafting. D.B. and R.A. contributed to data acquisition, and critically revised the manuscript. M.S. contributed to study design, data analysis and interpretation, and critically revised the manuscript.

## Conflicts of Interest

The authors declare no conflicts of interest related to this study.

## Supporting information


Tables S1–S2.

## Data Availability

The data of this study are available from the corresponding author upon reasonable request.

## Appendix A

**TABLE A1:** Patient‐level risk/protective indicators associated with incidence of peri‐implantitis during follow‐up: multilevel simple logistic regression analysis.

Variable	OR	95% CI	*p*
Age (years) (each unit increase) (baseline)	1.06	0.97–1.17	0.183
Age ≧ 65 years (yes) (baseline)	1.75	0.48–6.47	0.399
Gender (female) (baseline)	2.37	0.56–10.00	0.239
Educational level (baseline)			
Primary school	Ref	Ref	Ref
High school	1.17	0.25–5.51	0.840
Middle grade	0.31	0.04–2.47	0.269
University/College	0.19	0.02–1.56	0.121
Marital status (baseline)			
Married	Ref	Ref	Ref
Widow	7.27	1.05–50.48	**0.045**
Divorced	0.28	0.022–3.59	0.330
Never married	1.51	0.16–14.36	0.719
Living with unmarried partner	1.64	0.75–35.99	0.753
Height (cm) (each unit increase) (baseline)	0.92	0.85–0.98	**0.017**
Weight (kg) (each unit increase) (baseline)	0.96	0.92–1.01	0.120
BMI (kg/m^2^) (each unit increase) (baseline)	0.95	0.79–1.14	0.578
Diabetes* (yes) (baseline)	1.65	0.32–8.63	0.552
Osteoporosis/Osteopenia (yes) (baseline)	3.49	0.82–14.81	**0.090**
Myocardial Infarction (yes) (baseline)	11.08	0.156–787.56	0.269
Hypertension* (yes) (baseline)	1.30	0.317–5.29	0.718
Stroke (yes) (baseline)	5.58	0.14–229‐47	0.365
Anemia (yes) (baseline)	0.51	0.08–3.17	0.467
Cancer (yes) (baseline)	0.99	0.11–8.60	0.996
Depression* (yes) (baseline)	2.05	0.45–9.21	0.351
Asthma (yes) (baseline)	0.90	0.04–21.16	0.950
Cognitive or memory disorders (yes) (baseline)	1.10	0.07–17.77	0.945
Neurological disorders (yes) (baseline)	NE	NE	NE
Immunological disorders (yes) (baseline)	NE	NE	NE
Hypercholesterolemia (yes) (baseline)	0.80	0.21–3.09	0.745
Hepatitis (yes) (baseline)	0.43	0.02–0.12	**0.000**
HIV/AIDS (yes) (baseline)	NE	NE	NE
Rheumatoid arthritis (yes) (baseline)	2.59	0.49–13.79	0.264
Respiratory diseases (yes) (baseline)	0.64	0.071–5.77	0.692
Liver diseases (yes) (baseline)	NE	NE	NE
Cardiovascular diseases (yes) (baseline)	0.46	0.04–5.34	0.538
Gastrointestinal disorders (yes) (baseline)	1.38	0.27–7.15	0.699
Kidney diseases (yes) (baseline)	1.19	0.86–16.35	0.899
Thyroid disorders (yes) (baseline)	2.26	0.51–10.08	0.285
Cataract (yes) (baseline)	1.67	0.37–7.55	0.507
Other medical diseases (yes) (baseline)	0.58	0.7–5.01	0.618
Sars‐CoV 2 history (yes) (follow‐up)	0.78	0.20–3.03	0.725
Covid‐19 history (yes) (follow‐up)	0.53	0.13–2.20	0.383
Sars‐CoV 2 vaccine (yes) (follow‐up)	NE	NE	NE
At least one systemic disease (yes) (baseline)	0.39	0.73–2.07	0.269
Number of systemic diseases (each unit increase) (baseline)	1.12	0.86–1.45	0.394
Smoking (baseline)			
Non‐smokers	Ref	Ref	Ref
Current smokers	4.48	1.05–19.10	**0.043**
Sleep duration (baseline)			
< 7 h	1.97	0.56–6.95	0.290
7–8 h	Ref	Ref	Ref
> 7 h	50.91	1.46–1770.05	**0.030**
Regular moderate (≥ 3 times/week ≥ 20 min) physical activity (baseline)	1.35	0.23–7.94	0.741
Stress (baseline)			
Absolutely nothing	Ref	Ref	Ref
Mild/Moderate	0.64	0.13–3.14	0.581
High	0.97	0.11–8.77	0.979
Coffee consumption (yes) (baseline)	1.73	0.31–9.57	0.529
Alcohol consumption (baseline)			
Never	Ref	Ref	Ref
Less than 2 times/week	0.24	0.60–0.94	**0.040**
Almost everyday	0.46	0.07–3.17	0.432
1/day	0.24	0.25–2.32	0.217
2 or more times/day	NE	NE	NE
Alcohol consumption (yes) (baseline)			
Never	Ref	Ref	Ref
At least sometimes	0.26	0.78–0.88	**0.030**
Bisphosphonates (yes) (baseline)	0.04	0.01–0.12	**0.000**
Corticosteroids (yes) (baseline)	0.84	0.04–19.18	0.911
NSAIDs (yes) (baseline)	0.50	0.03–9.98	0.652
Antiplatelet (yes) (baseline)	2.14	0.20–22.60	0.528
Anticoagulant (yes) (baseline)	2.13	0.20–22.60	0.528
Hypolipidemic agent (yes) (baseline)	2.33	0.56–9.70	0.244
Antidepressant (yes) (baseline)	2.21	0.43–11.38	0.341
Proton pump inhibitor (yes) (baseline)	1.91	0.13–27.02	0.633
Vitamin D (yes) (baseline)	0.04	0.02–0.12	**0.000**
Calcium (yes) (baseline)	2.82	0.45–17.82	0.446
Thyroid drug (yes) (baseline)	2.49	0.55–11.28	0.236
Immunosuppressant (yes) (baseline)	0.41	0.01–0.12	**0.000**
Insulin (yes) (baseline)	3.02	0.22–40.70	0.406
Oral hypoglycemic agent (yes) (baseline)	1.31	0.18–9.57	0.790
Other anti‐diabetic drug (yes) (baseline)	NE	NE	NE
Beta‐blockers (yes) (baseline)	1.48	0.13–16.75	0.750
Diuretics (yes) (baseline)	0.92	0.09–9.36	0.944
ACE inhibitors (yes) (baseline)	1.56	0.21–11.65	0.666
Other antihypertensive drugs (yes) (baseline)	NE	NE	NE
Other drugs (yes) (baseline)	1.00	0.24–4.11	0.996
Number of medications (each unit increase) (baseline)	1.18	0.86–1.61	0.312
Supplements (yes) (baseline)	1.58	0.34–7.33	0.561
Periodontal status (AAP) (baseline)			
No/Mild periodontitis	Ref	Ref	Ref
Moderate/severe periodontitis	0.80	1.78–3.60	0.771
Edentulous	0.58	0.04–8.33	0.686
Periodontal status (2017 WWP) (baseline)			
No periodontitis or SI‐III periodontitis	Ref	Ref	Ref
Stage 4 periodontitis	9.08	2.58–31.92	**0.001**
Edentulous	1.42	0.19–10.69	0.736
Number of remaining teeth (each unit increase) (baseline)	0.91	0.83–0.99	**0.044**
Number of remaining teeth (≥ 16) (baseline)	9.68	2.56–36.57	**0.001**
Number of dental implants (each unit increase) (baseline)	1.24	0.95–1.63	0.120
Number of dental implants (≥ 4) (baseline)	4.14	0.63–27.06	0.139
History of orthodontic treatment (yes) (baseline)	1.20	0.30–4.75	0.795
Toothbrushing frequency (baseline)			
Not everyday	NE	NE	NE
1 time/day	1.68	0.22–12.89	0.615
2 times/day	1.24	0.30–5.09	0.766
3 or more times/day	Ref	Ref	Ref
Electric toothbrush (yes) (baseline)	4.68	1.00–21.96	**0.050**
Interproximal flossing/Brushing on implants (at least on some implants) (baseline)	4.59	0.31–67.48	0.267
Bruxism signs (yes) (baseline)	1.19	0.29–4.80	0.809
Bruxism symptoms (yes) (baseline)	2.03	0.46–8.92	0.346
Dry mouth (yes) (baseline)	2.50	0.66–9.51	0.177
Number of maintenances between baseline and follow‐up (each unit increase) (follow up)	1.15	0.70–1.87	0.584
Regular maintenance between baseline and follow‐up (≥ 1 per year) (follow up)	0.75	0.11–5.12	0.768
FMPS, excluding implants (each unit increase) (follow‐up)	1.01	0.95–1.06	0.810
FMBS, excluding implants (each unit increase) (follow‐up)	1.00	0.94–1.06	0.997
Number PPD ≥ 4 mm, excluding implants (each unit increase) (follow‐up)	1.00	0.95–1.06	0.885
Number PPD ≥ 5 mm, excluding implants (each unit increase) (follow‐up)	1.04	0.95–1.13	0.403
Number PPD ≥ 6 mm, excluding implants (each unit increase) (follow‐up)	1.10	0.96–1.26	0.181
Number FI ≥ 2 (each unit increase) (follow‐up)	1.16	0.75–1.77	0.509
Periodontal bone loss/age ratio (each unit increase) (follow‐up)	12.58	0.94–167.61	**0.055**
Allergies (yes) (baseline)	2.32	0.52–10.26	0.268
Chemotherapy (yes) (baseline)	5.58	0.14–229.47	0.365
Radiotherapy (yes) (baseline)	NE	NE	NE

*Note: p* < 0.10 are reported in bold.

Abbreviations: CI, confidence interval; FI, furcation involvement; FMBS, full mouth bleeding score; FMPS, full mouth plaque score; NE, not estimable; OR, odds ratio; PPD, probing pocket depth; Ref, reference category.

* Self‐reported history or medication.

**TABLE A2:** Implant‐level risk/protective indicators associated with incidence of peri‐implantitis during follow‐up: multilevel simple logistic regression analysis.

Variable	OR	95% CI	*p*
Jaw (maxilla) (baseline)	1.47	0.53–4.06	0.454
Position (baseline)			
Anterior (canine‐canine)	Ref	Ref	Ref
Posterior	0.61	0.17–2.23	0.434
Side (left) (baseline)	1.70	0.68–4.26	0.261
Replaced tooth (baseline)			
Molar	Ref	Ref	Ref
Premolar	1.06	0.37–3.00	0.914
Canine	NE	NE	NE
Incisor	5.48	1.07–28.04	**0.041**
Mouth zone (baseline)			
Posterior maxilla	Ref	Ref	Ref
Anterior maxilla	1.04	0.18–6.13	0.963
Posterior mandible	1.27	0.41–3.87	0.679
Anterior mandible	4.25	0.66–27.54	0.129
Implant brand (baseline)			
Straumann	Ref	Ref	Ref
Nobel biocare	2.56	0.43–15.27	0.304
Astratech	2.11	0.47–9,53	0.332
Other	1.10	0.26–4.73	0.894
Implant collar (baseline)			
0 mm	Ref	Ref	Ref
≤ 1.5 mm	0.61	0.50–7.36	0.693
> 1.5 mm	0.68	0.19–2.45	0.560
Implant length (each mm increase) (baseline)	0.94	0.68–1.30	0.690
Implant diameter (each mm increase) (baseline)	0.35	0.08–1.43	0.143
At least one adjacent tooth (yes) (baseline)	0.30	0.11–0.82	**0.019**
Reason of tooth loss (baseline)			
Caries	Ref	Ref	Ref
Periodontitis	1.19	0.31–4.57	0.803
Trauma	NE	NE	NE
Agenesia	NE	NE	NE
Other reason/Unknown	NE	NE	NE
Keratinized tissue width (KTW) (baseline)			
KTW = 0 mm	Ref	Ref	Ref
KTW > 0 mm & < =2 mm	0.38	0.10–1.43	0.153
KTW > 2 mm	1.02	0.25–4.12	0.983
Adherent mucosa (yes) (baseline)	1.22	0.42–3.58	0.715
Tissue thickness (mm) (baseline)	0.79	0.38–1.67	0.536
Peri‐implant phenotype (thick) (baseline)	0.83	0.30–2.28	0.711
Mucosal margin mobility (yes) (baseline)	0.60	0.20–1.83	0.370
Type of restoration (baseline)			
Single crown	Ref	Ref	Ref
Bridge	3.06	0.83–11.27	**0.093**
Overdenture	NE	NE	NE
Full‐arch fixed restoration	12.48	1.13–137.67	**0.039**
Restoration retention (baseline)			
Screw‐retained	Ref	Ref	Ref
Cemented	0.68	0.21–2.19	0.520
Locator	NE	NE	NE
Bar	NE	NE	NE
Prosthesis gap (yes) (baseline)	1.31	0.46–3.71	0.607
Prosthesis step (yes) (baseline)	2.25	0.80–6.35	0.126
Emergence angle (Highest) (each degree increase) (baseline)	1.01	0.98–1.03	0.707
Emergence angle (Highest) (> 30°) (baseline)	1.74	0.58–5.24	0.322
Emergence Profile (Worst) (baseline)			
Concave	NE	NE	NE
Straight	Ref	Ref	Ref
Convex	0.67	0.23–1.92	0.458
Mesial cantilever (yes) (baseline)	1.23	0.38–3.94	0.726
Distal cantilever (yes) (baseline)	0.52	0.12–2.24	0.377
Prosthesis mobility (yes) (baseline)	0.52	0.05–5.93	0.601
Abutment (yes) (baseline)	2.51	0.86–7.32	**0.093**
Platform switching (yes) (baseline)	1.13	0.31–4.09	0.854
Crown dimension (each mm increase) (baseline)	0.97	0.77–1.23	0.805
Crown to implant ratio (each mm increase) (baseline)	0.65	0.09–4.72	0.675
Residual cement visible on radiograph (yes) (baseline)	NE	NE	NE
Clinical signs of occlusal overloading (yes) (baseline)	1.16	0.37–3.59	0.799
Prosthetic design allowing access to hygiene (yes) (baseline)	0.21	0.059–0.73	**0.014**
Restoration margin location (follow‐up)			
Sub‐marginal	Ref	Ref	Ref
Supra‐marginal	0.61	0.063–5.81	0.664
Juxta‐marginal	2.87	0.87–9.48	**0.085**
Vestibular‐lingual position (baseline)			
Correct	Ref	Ref	Ref
Too vestibular	4.12	0.87–19.49	**0.074**
Too lingual	NE	NE	NE
Plaque (baseline)			
0–5 sites/implant	Ref	Ref	Ref
6 sites/implant	6.04	1.64–22.18	**0.007**
Peri‐implant health status (baseline)			
Peri‐implant health	Ref	Ref	Ref
Peri‐implant mucositis	1.76	0.16–19.82	0.646
Pre‐peri‐implantitis	1.08	0.94–12.58	0.946
Peri‐implantitis	2.34	0.20–27.13	0.498

*Note: p* < 0.10 are reported in bold.

Abbreviations: CI, confidence interval; OR, odds ratio; Ref, reference category.
