# Supplementary material for: Incidence and Risk Factors of Peri‐Implantitis Over Time—A Prospective Cohort Study
Source: J Periodontal Res. 2025 Jan 13;60(12):1222–36. doi: 10.1111/jre.13367 (PMC12881879; doi:10.1111/jre.13367)
Supplement: Supplementary file 1 — Tables S1–S2. [file JRE-60-1222-s001.docx]

**Table S1.** Descriptive statistics of the putative patient-level risk/protective factors, overall and according to incidence of peri-implantitis (N=72 patients).

| **Variable** | **Overall** | **Incidence of peri-implantitis** | |
| --- | --- | --- | --- |
|  |  | **No** | **Yes** |
| **Age (years),** (baseline), mean (SD) | 62.58 (7.84) | 61.54 (7.66) | 66.25 (7.59) |
| Missing, N (%) | 0 (0.0) | 0 (0.0) | 0 (0.0) |
| **Age ≧ 65 years** **(yes)** (baseline), N (%) |  |  |  |
| No | 42 (58.3) | 35 (62.5) | 7 (43.8) |
| Yes | 30 (41.7) | 21 (37.5) | 9 (56.3) |
| Missing | 0 (0.0) | 0 (0.0) | 0 (0.0) |
| **Gender (female)** (baseline), N (%) |  |  |  |
| Male | 27 (37.5) | 23 (41.1) | 4 (25.0) |
| Female | 45 (62.5) | 33 (58.9) | 12 (75.0) |
| Missing | 0 (0.0) | 0 (0.0) | 0 (0.0) |
| **Educational Level** (baseline), N (%) |  |  |  |
| Primary school | 22 (30.6) | 15 (26.8) | 7 (43.8) |
| High school | 17 (23.6) | 11 (19.6) | 6 (37.5) |
| Middle grade | 15 (20.8) | 14 (25.0) | 1 (6.2) |
| University/College | 18 (25.0) | 16 (28.6) | 2 (12.5) |
| Missing | 0 (0.0) | 0 (0.0) | 0 (0.0) |
| **Marital Status** (baseline), N (%) |  |  |  |
| Married | 53 (73.6) | 42 (75.0) | 11 (68.8) |
| Widow | 4 (5.6) | 2 (3.6) | 2 (12.5) |
| Divorced | 8 (11.1) | 7 (12.5) | 1 (6.2) |
| Never married | 5 (6.9) | 4 (7.1) | 1 (6.2) |
| Living with unmarried partner | 2 (2.8) | 1 (1.8) | 1 (6.2) |
| Missing | 0 (0.0) | 0 (0.0) | 0 (0.0) |
| **Height (cm)** (baseline), mean (SD) | 164.42 (8.50) | 165.29 (7.88) | 161.38 (10.10) |
| Missing, N (%) | 0 (0.0) | 0 (0.0) | 0 (0.0) |
| **Weight (kg)** (baseline), mean (SD) | 70.26 (14.81) | 70.94 (14.95) | 67.88 (14.51) |
| Missing, N (%) | 0 (0.0) | 0 (0.0) | 0 (0.0) |
| **BMI (kg/m²)** (baseline), mean (SD) | 25.80 (3.75) | 25.77 (3.78) | 25.88 (3.77) |
| Missing, N (%) | 0 (0.0) | 0 (0.0) | 0 (0.0) |
| **Diabetes*** (baseline), N (%) |  |  |  |
| No | 63 (87.5) | 51 (91.1) | 12 (75.0) |
| Yes | 9 (12.5) | 5 (8.9) | 4 (25.0) |
| Missing | 0 (0.0) | 0 (0.0) | 0 (0.0) |
| **Osteoporosis/Osteopenia** (baseline), N (%) |  |  |  |
| No | 58 (80.6) | 49 (87.5) | 9 (56.3) |
| Yes | 14 (19.4) | 7 (12.5) | 7 (43.7) |
| Missing | 0 (0.0) | 0 (0.0) | 0 (0.0) |
| **Myocardial Infarction** (baseline), N (%) |  |  |  |
| No | 71 (98.6) | 56 (100.0) | 15 (93.6) |
| Yes | 1 (1.4) | 0 (0.0) | 1 (6.4) |
| Missing | 0 (0.0) | 0 (0.0) | 0 (0.0) |
| **Hypertension*** (baseline), N (%) |  |  |  |
| No | 55 (76.4) | 46 (82.1) | 9 (56.2) |
| Yes | 17 (23.6) | 10 (17.9) | 7 (43.8) |
| Missing | 0 (0.0) | 0 (0.0) | 0 (0.0) |
| **Stroke** (baseline), N (%) |  |  |  |
| No | 71 (98.6) | 56 (100.0) | 15 (93.6) |
| Yes | 1 (1.4) | 0 (0.0) | 1 (6.4) |
| Missing | 0 (0.0) | 0 (0.0) | 0 (0.0) |
| **Anemia** (baseline), N (%) |  |  |  |
| No | 57 (79.2) | 43 (76.8) | 14 (87.5) |
| Yes | 15 (20.8) | 13 (23.2) | 2 (12.5) |
| Missing | 0 (0.0) | 0 (0.0) | 0 (0.0) |
| **Cancer** (baseline), N (%) |  |  |  |
| No | 66 (91.7) | 52 (92.9) | 14 (87.5) |
| Yes | 6 (8.3) | 4 (7.1) | 2 (12.5) |
| Missing | 0 (0.0) | 0 (0.0) | 0 (0.0) |
| **Depression***  (baseline), N (%) |  |  |  |
| No | 61 (84.7) | 48 (85.7) | 13 (81.3) |
| Yes | 11 (15.3) | 8 (14.3) | 3 (18.7) |
| Missing | 0 (0.0) | 0 (0.0) | 0 (0.0) |
| **Asthma** (baseline), N (%) |  |  |  |
| No | 68 (94.4) | 53 (94.6) | 15 (93.8) |
| Yes | 4 (5.6) | 3 (5.4) | 1 (6.2) |
| Missing | 0 (0.0) | 0 (0.0) | 0 (0.0) |
| **Cognitive or Memory Disorders** (baseline), N (%) |  |  |  |
| No | 69 (95.8) | 54 (96.4) | 15 (93.8) |
| Yes | 3 (4.2) | 2 (3.6) | 1 (6.2) |
| Missing | 0 (0.0) | 0 (0.0) | 0 (0.0) |
| **Neurological Disorders** (baseline), N (%) |  |  |  |
| No | 69 (95.8) | 53 (94.6) | 16 (100.0) |
| Yes | 3 (4.2) | 3 (5.4) | 0 (0.0) |
| Missing | 0 (0.0) | 0 (0.0) | 0 (0.0) |
| **Immunological Disorders** (baseline), N (%) |  |  |  |
| No | 71 (98.6) | 55 (98.2) | 16 (100.0) |
| Yes | 1 (1.4) | 1 (1.8) | 0 (0.0) |
| Missing | 0 (0.0) | 0 (0.0) | 0 (0.0) |
| **Hypercholesterolemia** (baseline), N (%) |  |  |  |
| No | 44 (61.1) | 34 (60.7) | 10 (62.5) |
| Yes | 28 (38.9) | 22 (39.3) | 6 (37.5) |
| Missing | 0 (0.0) | 0 (0.0) | 0 (0.0) |
| **Hepatitis** (baseline), N (%) |  |  |  |
| No | 70 (97.2) | 54 (96.4) | 16 (100.0) |
| Yes | 2 (2.8) | 2 (3.6) | 0 (0.0) |
| Missing | 0 (0.0) | 0 (0.0) | 0 (0.0) |
| **HIV/AIDS** (baseline), N (%) |  |  |  |
| No | 72 (100.0) | 56 (100.0) | 16 (100.0) |
| Yes | 0 (0.0) | 0 (0.0) | 0 (0.0) |
| Missing | 0 (0.0) | 0 (0.0) | 0 (0.0) |
| **Rheumatoid Arthritis** (baseline), N (%) |  |  |  |
| No | 60 (83.3) | 49 (87.5) | 11 (68.8) |
| Yes | 12 (16.7) | 7 (12.5) | 5 (31.2) |
| Missing | 0 (0.0) | 0 (0.0) | 0 (0.0) |
| **Respiratory Diseases** (baseline), N (%) |  |  |  |
| No | 64 (88.9) | 50 (89.3) | 14 (87.5) |
| Yes | 8 (11.1) | 6 (10.7) | 2 (12.5) |
| Missing | 0 (0.0) | 0 (0.0) | 0 (0.0) |
| **Liver Diseases** (baseline), N (%) |  |  |  |
| No | 69 (95.8) | 53 (94.6) | 16 (100.0) |
| Yes | 3 (4.2) | 3 (5.4) | 0 (0.0) |
| Missing | 0 (0.0) | 0 (0.0) | 0 (0.0) |
| **Cardiovascular Diseases** (baseline), N (%) |  |  |  |
| No | 65 (90.3) | 50 (89.3) | 15 (93.8) |
| Yes | 7 (9.7) | 6 (10.7) | 1 (6.2) |
| Missing | 0 (0.0) | 0 (0.0) | 0 (0.0) |
| **Gastrointestinal Disorders** (baseline), N (%) |  |  |  |
| No | 58 (80.6) | 46 (82.1) | 12 (75.0) |
| Yes | 14 (19.4) | 10 (17.9) | 4 (25.0) |
| Missing | 0 (0.0) | 0 (0.0) | 0 (0.0) |
| **Kidney Diseases** (baseline), N (%) |  |  |  |
| No | 68 (94.4) | 53 (94.6) | 15 (93.8) |
| Yes | 4 (5.6) | 3 (5.4) | 1 (6.2) |
| Missing | 0 (0.0) | 0 (0.0) | 0 (0.0) |
| **Thyroid Disorders** (baseline), N (%) |  |  |  |
| No | 59 (81.9) | 47 (83.9) | 12 (75.0) |
| Yes | 13 (18.1) | 9 (16.1) | 4 (25.0) |
| Missing | 0 (0.0) | 0 (0.0) | 0 (0.0) |
| **Cataract** (baseline), N (%) |  |  |  |
| No | 58 (80.6) | 48 (85.7) | 10 (62.5) |
| Yes | 14 (19.4) | 8 (14.3) | 6 (37.5) |
| Missing | 0 (0.0) | 0 (0.0) | 0 (0.0) |
| **Other Medical Diseases** (baseline), N (%) |  |  |  |
| No | 62 (86.1) | 48 (85.7) | 14 (87.5) |
| Yes | 10 (13.9) | 8 (14.3) | 2 (12.5) |
| Missing | 0 (0.0) | 0 (0.0) | 0 (0.0) |
| **Sars-CoV 2 History**  (follow-up), N (%) |  |  |  |
| No | 30 (41.7) | 21 (37.5) | 9 (56.3) |
| Yes | 42 (58.3) | 35 (62.5) | 7 (43.7) |
| Missing | 0 (0.0) | 0 (0.0) | 0 (0.0) |
| **Covid-19 History** (follow-up), N (%) |  |  |  |
| No | 36 (50.0) | 25 (44.6) | 11 (68.8) |
| Yes | 36 (50.0) | 31 (53.4) | 5 (31.2) |
| Missing | 0 (0.0) | 0 (0.0) | 0 (0.0) |
| **Sars-CoV 2 vaccine** (follow-up), N (%) |  |  |  |
| No | 1 (1.4) | 1 (1.8) | 0 (0.0) |
| Yes | 71 (98.6) | 55 (98.2) | 16 (100.0) |
| Missing | 0 (0.0) | 0 (0.0) | 0 (0.0) |
| **At least one systemic disease** (baseline), N (%) |  |  |  |
| No | 11 (15.3) | 8 (14.3) | 3 (18.7) |
| Yes | 61 (84.7) | 48 (85.7) | 13 (81.3) |
| Missing | 0 (0.0) | 0 (0.0) | 0 (0.0) |
| **Number of systemic diseases** (baseline), mean (SD) | 2.78 (2.50) | 2.5 (2.45) | 3.75 (2.49) |
| Missing, N (%) | 0 (0.0) | 0 (0.0) | 0 (0.0) |
| **Smoking** (baseline), N (%) |  |  |  |
| Non-smokers | 58 (80.6) | 46 (82.1) | 12 (75.0) |
| Current smokers | 14 (19.4) | 10 (17.9) | 4 (25.0) |
| Missing | 0 (0.0) | 0 (0.0) | 0 (0.0) |
| **Sleep Duration** (baseline), N (%) |  |  |  |
| <7 hours | 29 (40.3) | 20 (35.7) | 9 (56.3) |
| 7-8 hours | *42 (58.3)* | 36 (64.3) | 6 (37.5) |
| >7 hours | 1 (1.4) | 0 (0.0) | 1 (6.2) |
| Missing | 0 (0.0) | 0 (0.0) | 0 (0.0) |
| **Regular Moderate (≥3 times/week ≥20 minutes) Physical Activity** (baseline), N (%) |  |  |  |
| No | 14 (19.4) | 11 (19.6) | 3 (18.7) |
| Yes | 58 (80.6) | 45 (80.4) | 13 (81.3) |
| Missing | 0 (0.0) | 0 (0.0) | 0 (0.0) |
| **Stress** (baseline), N (%) |  |  |  |
| Absolutely nothing | 16 (22.2) | 13 (23.2) | 3 (18.8) |
| Mild / Moderate | 47 (65.3) | 37 (66.1) | 10 (62.4) |
| High | 9 (12.5) | 6 (10.7) | 3 (18.8) |
| Missing | 0 (0.0) | 0 (0.0) | 0 (0.0) |
| **Coffee Consumption** (baseline), N (%) |  |  |  |
| No | 15 (20.8) | 12 (21.4) | 3 (18.8) |
| Yes | 57 (79.2) | 44 (78.6) | 13 (81.2) |
| Missing | 0 (0.0) | 0 (0.0) | 0 (0.0) |
| **Alcohol Consumption** (baseline), N (%) |  |  |  |
| Never | 23 (32.0) | 15 (26.8) | 8 (50.0) |
| Less than 2 times/week | 33 (45.8) | 28 (50.0) | 5 (31.3) |
| Almost everyday | 8 (11.1) | 6 (10.7) | 2 (12.5) |
| 1/day | 6 (8.3) | 5 (8.9) | 1 (6.2) |
| 2 or more times/day | 2 (2.8) | 2 (3.6) | 0 (0.0) |
| Missing | 0 (0.0) | 0 (0.0) | 0 (0.0) |
| **Alcohol Consumption)** (baseline), N (%) |  |  |  |
| Never | 23 (31.9) | 15 (26.8) | 8 (50.0) |
| At least sometimes | 49 (68.1) | 41 (73.2) | 8 (50.0) |
| Missing | 0 (0.0) | 0 (0.0) | 0 (0.0) |
| **Bisphosphonates** (baseline), N (%) |  |  |  |
| No | 72 (100.0) | 56 (100.0) | 16 (100.0) |
| Yes | 0 (0.0) | 0 (0.0) | 0 (0.0) |
| Missing | 0 (0.0) | 0 (0.0) | 0 (0.0) |
| **Corticosteroids)** (baseline), N (%) |  |  |  |
| No | 68 (94.4) | 53 (94.6) | 15 (93.8) |
| Yes | 4 (5.6) | 3 (5.4) | 1 (6.2) |
| Missing | 0 (0.0) | 0 (0.0) | 0 (0.0) |
| **NSAIDs** (baseline), N (%) |  |  |  |
| No | 68 (94.4) | 53 (94.6) | 15 (93.8) |
| Yes | 4 (5.6) | 3 (5.4) | 1 (6.2) |
| Missing | 0 (0.0) | 0 (0.0) | 0 (0.0) |
| **Antiplatelet** (baseline), N (%) |  |  |  |
| No | 68 (94.4) | 54 (96.4) | 14 (87.5) |
| Yes | 4 (5.6) | 2 (3.6) | 2 (12.5) |
| Missing | 0 (0.0) | 0 (0.0) | 0 (0.0) |
| **Anticoagulant** (baseline), N (%) |  |  |  |
| No | 68 (94.4) | 54 (96.4) | 14 (87.5) |
| Yes | 4 (5.6) | 2 (3.6) | 2 (12.5) |
| Missing | 0 (0.0) | 0 (0.0) | 0 (0.0) |
| **Hypolipidemic agent** (baseline), N (%) |  |  |  |
| No | 56 (77.8) | 46 (82.1) | 10 (62.5) |
| Yes | 16 (22.2) | 10 (17.9) | 6 (37.5) |
| Missing | 0 (0.0) | 0 (0.0) | 0 (0.0) |
| **Antidepressant** (baseline), N (%) |  |  |  |
| No | 61 (84.7) | 49 (87.5) | 12 (75.0) |
| Yes | 11 (15.3) | 7 (12.5) | 4 (12.5) |
| Missing | 0 (0.0) | 0 (0.0) | 0 (0.0) |
| **Proton Pump Inhibitor** (baseline), N (%) |  |  |  |
| No | 69 (95.8) | 55 (98.2) | 14 (87.5) |
| Yes | 3 (4.2) | 1 (1.8) | 2 (12.5) |
| Missing | 0 (0.0) | 0 (0.0) | 0 (0.0) |
| **Vitamin D** (baseline), N (%) |  |  |  |
| No | 69 (95.8) | 43 (94.6) | 16 (100.0) |
| Yes | 3 (4.2) | 3 (5.4) | 0 (0.0) |
| Missing | 0 (0.0) | 0 (0.0) | 0 (0.0) |
| **Calcium**(baseline), N (%) |  |  |  |
| No | 65 (90.3) | 53 (94.6) | 12 (75.0) |
| Yes | 7 (9.7) | 3 (5.4) | 4 (25.0) |
| Missing | 0 (0.0) | 0 (0.0) | 0 (0.0) |
| **Thyroid Drug** (baseline), N (%) |  |  |  |
| No | 60 (93.3) | 48 (85.7) | 12 (75.0) |
| Yes | 12 (16.7) | 8 (14.3) | 4 (25.0) |
| Missing | 0 (0.0) | 0 (0.0) | 0 (0.0) |
| **Immunosuppressant** (baseline), N (%) |  |  |  |
| No | 71 (98.6) | 55 (98.2) | 16 (100.0) |
| Yes | 1 (1.4) | 1 (1.8) | 0 (0.0) |
| Missing | 0 (0.0) | 0 (0.0) | 0 (0.0) |
| **Insulin** (baseline), N (%) |  |  |  |
| No | 69 (95.8) | 54 (96.4) | 15 (93.8) |
| Yes | 3 (4.2) | 2 (3.6) | 1 (6.3) |
| Missing | 0 (0.0) | 0 (0.0) | 0 (0.0) |
| **Oral Hypoglycemic Agent** (baseline), N (%) |  |  |  |
| No | 65 (90.3) | 55 (91.1) | 14 (87.5) |
| Yes | 7 (9.7) | 5 (8.9) | 2 (12.5) |
| Missing | 0 (0.0) | 0 (0.0) | 0 (0.0) |
| **Other Anti-diabetic Drug** (baseline), N (%) |  |  |  |
| No | 70 (97.2) | 54 (96.4) | 16 (100.0) |
| Yes | 2 (2.8) | 2 (3.6) | 0 (0.0) |
| Missing | 0 (0.0) | 0 (0.0) | 0 (0.0) |
| **Beta-blockers** (baseline), N (%) |  |  |  |
| No | 68 (94.4) | 53 (94.6) | 15 (93.8) |
| Yes | 4 (5.6) | 3 (5.4) | 1 (6.3) |
| Missing | 0 (0.0) | 0 (0.0) | 0 (0.0) |
| **Diuretics** (baseline), N (%) |  |  |  |
| No | 67 (93.1) | 52 (98.9) | 15 (93.8) |
| Yes | 5 (6.9) | 4 (7.1) | 1 (6.3) |
| Missing | 0 (0.0) | 0 (0.0) | 0 (0.0) |
| **ACE Inhibitors** (baseline), N (%) |  |  |  |
| No | 66 (91.7) | 53 (94.6) | 13 (81.3) |
| Yes | 6 (8.3) | 3 (5.4) | 3 (18.7) |
| Missing | 0 (0.0) | 0 (0.0) | 0 (0.0) |
| **Other Antihypertensive Drugs** (baseline), N (%) |  |  |  |
| No | 70 (97.2) | 54 (96.4) | 16 (100.0) |
| Yes | 2 (2.8) | 2 (3.6) | 0 (0.0) |
| Missing | 0 (0.0) | 0 (0.0) | 0 (0.0) |
| **Other Drugs** (baseline), N (%) |  |  |  |
| No | 51 (70.8) | 40 (71.4) | 11 (68.8) |
| Yes | 21 (29.2) | 16 (28.6) | 5 (31.2) |
| Missing | 0 (0.0) | 0 (0.0) | 0 (0.0) |
| **Number of medications** (baseline), mean (SD) | 1.65 (1.92) | 1.43 (1.82) | 2.44 (2.13) |
| Missing, N (%) | 0 (0.0) | 0 (0.0) | 0 (0.0) |
| **Supplements** (baseline), N (%) |  |  |  |
| No | 58 (80.6) | 46 (82.1) | 12 (75.0) |
| Yes | 14 (19.4) | 10 (17.9) | 4 (25.0) |
| Missing | 0 (0.0) | 0 (0.0) | 0 (0.0) |
| **Periodontal status (2017 WWP)** (baseline), N (%) |  |  |  |
| No periodontitis or SI-III periodontitis | 49 (68.1) | 43 (76.8) | 6 (37.5) |
| Stage 4 periodontitis | 17 (23.6) | 8 (14.3) | 9 (56.3) |
| Edentulous | 6 (8.3) | 5 (8.9) | 1 (6.2) |
| Missing | 0 (0.0) | 0 (0.0) | 0 (0.0) |
| **Number of Remaining Teeth** (baseline), mean (SD) | 18.89 (7.50) | 20.30 (7.28) | 13.94 (6.22) |
| Missing, N (%) | 0 (0.0) | 0 (0.0) | 0 (0.0) |
| **Number of Remaining Teeth (≥16)** (baseline), N (%) |  |  |  |
| No | 20 (27.8) | 9 (16.1) | 11 (68.8) |
| Yes | 52 (72.2) | 47 (83.9) | 5 (31.2) |
| Missing, N (%) | 0 (0.0) | 0 (0.0) | 0 (0.0) |
| **Number of Dental Implants** (baseline), mean (SD) | 4.51 (2.71) | 3.89 (2.44) | 6.69 (2.52) |
| Missing, N (%) | 0 (0.0) | 0 (0.0) | 0 (0.0) |
| **Number of Dental Implants (≥4)** (baseline), N (%) |  |  |  |
| No | 30 (41.7) | 28 (50.0) | 2 (12.5) |
| Yes | 42 (58.3) | 28 (50.0) | 14 (87.5) |
| Missing | 0 (0.0) | 0 (0.0) | 0 (0.0) |
| **History of Orthodontic Treatment** (baseline), N (%) |  |  |  |
| No | 50 (64.4) | 40 (71.4) | 10 (62.5) |
| Yes | 22 (33.6) | 16 (28.6) | 6 (37.5) |
| Missing | 0 (0.0) | 0 (0.0) | 0 (0.0) |
| **Toothbrushing Frequency** (baseline), N (%) |  |  |  |
| Not everyday | 1 (1.4) | 1 (1.8) | 0 (0.0) |
| 1 time/day | 7 (9.7) | 4 (7.1) | 3 (18.7) |
| 2 times/day | 31 (43.1) | 25 (44.7) | 6 (37.5) |
| 3 or more times/day | 33 (45.8) | 26 (46.4) | 7 (43.8) |
| Missing | 0 (0.0) | 0 (0.0) | 0 (0.0) |
| **Electric Toothbrush** (baseline), N (%) |  |  |  |
| No | 27 (37.5) | 24 (42.9) | 3 (18.7) |
| Yes | 45 (62.5) | 32 (57.1) | 13 (81.3) |
| Missing | 0 (0.0) | 0 (0.0) | 0 (0.0) |
| **Interproximal Flossing/Brushing on Implants (at least on some implants)** (baseline), N (%) |  |  |  |
| No | 8 (11.1) | 7 (12.5) | 1 (6.2) |
| Yes | 64 (88.9) | 49 (87.5) | 15 (93.8) |
| Missing | 0 (0.0) | 0 (0.0) | 0 (0.0) |
| **Bruxism Signs** (baseline), N (%) |  |  |  |
| No | 47 (65.3) | 36 (64.3) | 11 (68.8) |
| Yes | 25 (34.7) | 20 (35.7) | 5 (31.2) |
| Missing | 0 (0.0) | 0 (0.0) | 0 (0.0) |
| **Bruxism Symptoms** (baseline), N (%) |  |  |  |
| No | 56 (77.8) | 45 (80.4) | 11 (68.8) |
| Yes | 16 (22.2) | 11 (19.6) | 5 (31.2) |
| Missing | 0 (0.0) | 0 (0.0) | 0 (0.0) |
| **Dry Mouth** (baseline), N (%) |  |  |  |
| No | 51 (79.8) | 42 (75.0) | 9 (56.3) |
| Yes | 21 (29.2) | 14 (25.0) | 7 (43.7) |
| Missing | 0 (0.0) | 0 (0.0) | 0 (0.0) |
| **Number of maintenances between baseline and follow-up** (follow up), mean (SD) | 2.06 (1.47) | 1.95 (1.53) | 2.44 (1.21) |
| Missing, N (%) | 0 (0.0) | 0 (0.0) | 0 (0.0) |
| **Regular maintenance between baseline and follow-up (≥1 per year)** (follow up), N (%) |  |  |  |
| No | 33 (45.8) | 26 (46.4) | 7 (43.7) |
| Yes | 39 (54.2) | 30 (53.6) | 9 (56.3) |
| Missing | 0 (0.0) | 0 (0.0) | 0 (0.0) |
| **FMPS, excluding implants** (follow-up), mean (SD) | 33.47 (16.13) | 33.51 (16.47) | 33.32 (15.29) |
| Missing, N (%) | 9 (3.0) | 5 (1.8) | 4 (14.3) |
| **FMBS, excluding implants** (follow-up), mean (SD) | 19.86 (12.50) | 19.69 (12.44) | 20.54 (13.19) |
| Missing, N (%) | 8 (2.7) | 5 (1.9) | 3 (10.7) |
| **Number PPD≥4mm, excluding implants** (follow-up), mean (SD) | 16.54 (14.27) | 16.77 (14.01) | 15.62 (15.87) |
| Missing, N (%) | 7 (2.3) | 4 (1.5) | 3 (10.7) |
| **Number PPD≥5mm, excluding implants** (follow-up), mean (SD) | 5.83 (7.42) | 5.56 (6.89) | 6.92 (9.51) |
| Missing, N (%) | 7 (2.3) | 4 (1.5) | 3 (10.7) |
| **Number PPD≥6mm, excluding implants** (follow-up), mean (SD) | 2.38 (4.12) | 2.08 (3.20) | 3.62 (6.70) |
| Missing, N (%) | 7 (2.3) | 4 (1.5) | 3 (10.7) |
| **Number FI ≥ 2** (follow-up), mean (SD) | 0.83 (1.68) | 0.84 (1.63) | 0.77 (1.92) |
| Missing, N (%) | 9 (3.0) | 6 (2.2) | 3 (10.7) |
| **Periodontal bone loss/age ratio** (follow-up), mean (SD) | 0.65 (0.29) | 0.61 (0.27) | 0.78 (0.31) |
| Missing, N (%) | 10 (3.4) | 8 (3.0) | 2 (7.1) |
| **Allergies** (baseline), N (%) |  |  |  |
| No | 55 (76.4) | 45 (80.4) | 10 (62.5) |
| Yes | 17 (23.6) | 11 (19.6) | 6 (37.5) |
| Missing | 0 (0.0) | 0 (0.0) | 0 (0.0) |
| **Chemotherapy** (baseline), N (%) |  |  |  |
| No | 71 (93.6) | 56 (100.0) | 15 (93.8) |
| Yes | 1 (1.4) | 0 (0.0) | 1 (6.2) |
| Missing | 0 (0.0) | 0 (0.0) | 0 (0.0) |
| **Radiotherapy** (baseline), N (%) |  |  |  |
| No | 70 (97.2) | 54 (96.4) | 16 (100.0) |
| Yes | 2 (2.8) | 2 (3.6) | 0 (0.0) |
| Missing | 0 (0.0) | 0 (0.0) | 0 (0.0) |

*Footnote:*

FI, furcation involvement; FMBS, full mouth bleeding score; FMPS, full mouth plaque score; N, number; PPD, probing pocket depth; SD, standard deviation.

* self-reported history or medication

**Table S2.** Descriptive statistics of the putative implant-level risk/protective factors, overall and according to incidence of peri-implantitis (N=298 implants).

| **Variable** | **Overall** | **Incidence of peri-implantitis** | |
| --- | --- | --- | --- |
|  |  | **No** | **Yes** |
| **Jaw** (baseline), N (%) |  |  |  |
| Maxilla | 160 (53.7) | 150 (55.6) | 10 (35.7) |
| Mandible | 138 (46.3) | 120 (44.4) | 18 (64.3) |
| Missing | 0 (0.0) | 0 (0.0) | 0 (0.0) |
| **Position** (baseline), N (%) |  |  |  |
| Anterior (canine-canine) | 49 (16.4) | 44 (16.3) | 5 (17.9) |
| Posterior | 249 (85.6) | 226 (83.7) | 23 (82.1) |
| Missing | 0 (0.0) | 0 (0.0) | 0 (0.0) |
| **Side** (baseline), N (%) |  |  |  |
| Right | 152 (51.0) | 140 (51.9) | 12 (42.9) |
| Left | 146 (49.0) | 130 (48.1) | 16 (57.1) |
| Missing | 0 (0.0) | 0 (0.0) | 0 (0.0) |
| **Replaced tooth** (baseline), N (%) |  |  |  |
| Molar | 145 (48.7) | 132 (48.9) | 13 (46.4) |
| Premolar | 104 (34.9) | 94 (34.8) | 10 (35.7) |
| Canine | 20 (6.7) | 20 (7.4) | 0 (0.0) |
| Incisor | 29 (9.7) | 24 (8.9) | 5 (17.9) |
| Missing | 0 (0.0) | 0 (0.0) | 0 (0.0) |
| **Mouth zone** (baseline), N (%) |  |  |  |
| Posterior Maxilla | 127 (42.6) | 119 (44.1) | 8 (28.6) |
| Anterior Maxilla | 33 (11.1) | 31 (11.5) | 2 (7.1) |
| Posterior Mandible | 122 (40.9) | 107 (39.6) | 15 (53.6) |
| Anterior Mandible | 16 (5.4) | 13 (4.8) | 3 (10.7) |
| Missing | 0 (0.0) | 0 (0.0) | 0 (0.0) |
| **Implant Brand** (baseline), N (%) |  |  |  |
| Straumann, | 164 (55.0) | 150 (55.6) | 14 (50.0) |
| Nobel Biocare | 36 (12.1) | 31 (11.5) | 5 (17.9) |
| AstraTech | 40 (13.4) | 35 (13.0) | 5 (17.9) |
| Other | 57 (19.1) | 53 (19.6) | 4 (14.3) |
| Missing | 1 (0.4) | 1 (0.4) | 0 (0.0) |
| **Implant Collar** (baseline), N (%) |  |  |  |
| 0 mm | 88 (29.5) | 81 (30.0) | 7 (25.0) |
| ≤ 1.5 mm | 28 (9.4) | 27 (10.0) | 1 (3.6) |
| > 1.5 mm | 148 (49.7) | 139 (51.5) | 9 (32.1) |
| Missing | 34 (11.4) | 23 (8.5) | 11 (39.3) |
| **Implant length** (baseline), mean (SD) | 9.88 (1.68) | 9.92 (1.68) | 9.46 (1.61) |
| Missing, N (%) | 5 (1.7) | 4 (1.5) | 1 (3.6) |
| **Implant diameter** (baseline), mean (SD) | 4.14 (0.40) | 4.15 (0.40) | 4.04 (0.35) |
| Missing, N (%) | 3 (1.0) | 2 (0.7) | 1 (3.6) |
| **At Least One adjacent Tooth** (baseline), N (%) |  |  |  |
| No | 122 (40.9) | 104 (38.5) | 18 (64.3) |
| Yes | 176 (59.1) | 166 (61.5) | 10 (35.7) |
| Missing | 0 (0.0) | 0 (0.0) | 0 (0.0) |
| **Reason of Tooth Loss** (baseline), N (%) |  |  |  |
| Caries | 118 (39.6) | 107 (39.6) | 11 (39.3) |
| Periodontitis | 119 (39.9) | 107 (39.6) | 12 (42.8) |
| Trauma | 9 (3.0) | 9 (3.3) | 0 (0.0) |
| Agenesia | 4 (1.4) | 4 (1.5) | 0 (0.0) |
| Other reason/Unknown | 48 (16.1) | 43 (16.0) | 5 (17.9) |
| Missing | 0 (0.0) | 0 (0.0) | 0 (0.0) |
| **Keratinized Tissue Width (KTW)** (baseline), N (%) |  |  |  |
| KTW=0 mm | 49 (16.4) | 42 (15.6) | 7 (25.0) |
| KTW >0 mm & <=2mm | 125 (42.0) | 116 (42.9) | 9 (32.1) |
| KTW>2mm | 124 (41.6) | 112 (41.5) | 12 (42.9) |
| Missing | 0 (0.0) | 0 (0.0) | 0 (0.0) |
| **Adherent mucosa** (baseline), N (%) |  |  |  |
| No | 203 (68.1) | 183 (67.8) | 20 (71.4) |
| Yes | 95 (31.9) | 87 (32.2) | 8 (28.6) |
| Missing | 0 (0.0) | 0 (0.0) | 0 (0.0) |
| **Tissue Thickness** (baseline,) mean (SD) | 1.46 (0.74) | 1.47 (0.75) | 1.33 (0.67) |
| Missing, N (%) | 17 (5.7) | 17 (6.3) | 0 (0.0) |
| **Peri-implant phenotype** (baseline), N (%) |  |  |  |
| Thin | 111 (37.3) | 99 (36.7) | 12 (42.9) |
| Thick | 183 (61.4) | 167 (61.8) | 16 (57.1) |
| Missing | 4 (1.3) | 4 (1.5) | 0 (0.0) |
| **Mucosal Margin Mobility** (baseline), N (%) |  |  |  |
| No | 152 (51.0) | 137 (50.7) | 15 (53.6) |
| Yes | 114 (38.3) | 105 (38.9) | 9 (32.1) |
| Missing | 32 (10.7) | 28 (10.4) | 4 (14.3) |
| **Type of Restoration** (baseline), N (%) |  |  |  |
| Single crown | 98 (32.9) | 94 (34.8) | 4 (14.3) |
| Bridge | 174 (58.4) | 154 (57.0) | 20 (71.4) |
| Overdenture | 7 (2.4) | 7 (2.6) | 0 (0.0) |
| Full-arch fixed restoration | 19 (6.4) | 15 (5.6) | 4 (14.3) |
| Missing | 0 (0.0) | 0 (0.0) | 0 (0.0) |
| **Restoration Retention** (baseline), N (%) |  |  |  |
| Screw-Retained | 137 (45.9) | 125 (46.3) | 12 (42.9) |
| Cemented | 154 (51.7) | 138 (51.1) | 16 (57.1) |
| Locator | 2 (0.7) | 2 (0.7) | 0 (0.0) |
| Bar | 5 (1.7) | 5 (1.9) | 0 (0.0) |
| Missing | 0 (0.0) | 0 (0.0) | 0 (0.0) |
| **Prosthesis Gap** (baseline), N (%) |  |  |  |
| No | 219 (73.5) | 201 (74.4) | 18 (64.3) |
| Yes | 79 (26.5) | 69 (25.6) | 10 (35.7) |
| Missing | 0 (0.0) | 0 (0.0) | 0 (0.0) |
| **Prosthesis Step** (baseline), N (%) |  |  |  |
| No | 129 (43.3) | 120 (44.4) | 9 (32.1) |
| Yes | 169 (56.7) | 150 (55.6) | 19 (67.9) |
| Missing | 0 (0.0) | 0 (0.0) | 0 (0.0) |
| **Emergence Angle (Worst)** (baseline), mean (SD) | 41.54 (19.78) | 41.46 (20.05) | 42.26 (17.24) |
| Missing, N (%) | 4 (1.3) | 3 (1.1) | 1 (3.6) |
| **Emergence Angle (>30°)** (baseline), N (%) |  |  |  |
| No | 99 (32.2) | 92 (34.1) | 7 (25.0) |
| Yes | 195 (65.4) | 175 (64.8) | 20 (71.4) |
| Missing | 4 (1.3) | 3 (1.1) | 1 (3.6) |
| **Emergence Profile (Worst)** (baseline), N (%) |  |  |  |
| Concave | 13 (4.4) | 13 (4.8) | 0 (0.0) |
| Straight | 81 (27.2) | 72 (26.7) | 9 (32.2) |
| Convex | 199 (66.8) | 182 (67.4) | 17 (60.7) |
| Missing | 5 (1.7) | 3 (1.1) | 2 (7.1) |
| **Mesial Cantilever** (baseline), N (%) |  |  |  |
| No | 244 (81.9) | 222 (82.2) | 22 (78.6) |
| Yes | 49 (16.4) | 43 (15.9) | 6 (21.4) |
| Missing | 5 (1.7) | 5 (1.9) | 0 (0.0) |
| **Distal Cantilever** (baseline), N (%) |  |  |  |
| No | 243 (81.5) | 218 (80.7) | 25 (89.3) |
| Yes | 50 (16.8) | 47 (14.4) | 3 (10.7) |
| Missing | 5 (1.7) | 5 (1.9) | 0 (0.0) |
| **Prosthesis Mobility** (baseline), N (%) |  |  |  |
| No | 273 (91.6) | 246 (91.1) | 27 (96.4) |
| Yes | 25 (8.4) | 24 (8.9) | 1 (3.6) |
| Missing | 0 (0.0) | 0 (0.0) | 0 (0.0) |
| **Abutment** (baseline), N (%) |  |  |  |
| No | 212 (71.1) | 198 (73.3) | 14 (50.0) |
| Yes | 86 (28.9) | 72 (26.7) | 14 (50.0) |
| Missing | 0 (0.0) | 0 (0.0) | 0 (0.0) |
| **Platform Switching** (baseline), N (%) |  |  |  |
| No | 234 (78.5) | 212 (78.5) | 22 (78.6) |
| Yes | 64 (21.5) | 58 (21.5) | 6 (21.4) |
| Missing | 0 (0.0) | 0 (0.0) | 0 (0.0) |
| **Crown Dimension** (baseline), mean (SD) | 11.36 (2.34) | 11.42 (2.34) | 10.80 (2.30) |
| Missing, N (%) | 17 (5.7) | 15 (5.6) | 2 (7.1) |
| **Crown to Implant Ratio** (baseline), mean (SD) | 1.19 (0.33) | 1.19 (0.34) | 1.15 (0.22) |
| Missing, N (%) | 22 (7.4) | 19 (7.0) | 3 (10.7) |
| **Residual Cement Visible on Radiograph** (baseline), N (%) |  |  |  |
| No | 294 (98.7) | 266 (98.5) | 28 (100.0) |
| Yes | 4 (1.3) | 4 (1.5) | 0 (0.0) |
| Missing | 0 (0.0) | 0 (0.0) | 0 (0.0) |
| **Clinical Signs of Occlusal Overloading** (baseline), N (%) |  |  |  |
| No | 184 (61.7) | 167 (61.9) | 17 (60.7) |
| Yes | 114 (38.3) | 103 (38.1) | 11 (39.3) |
| Missing | 0 (0.0) | 0 (0.0) | 0 (0.0) |
| **Prosthetic design** **allowing access to hygiene** (baseline), N (%) |  |  |  |
| No | 52 (17.5) | 43 (15.9) | 9 (32.1) |
| Yes | 246 (82.6) | 227 (84.1) | 19 (67.9) |
| Missing | 0 (0.0) | 0 (0.0) | 0 (0.0) |
| **Restoration margin location** (follow-up), N (%) |  |  |  |
| Sub-marginal | 231 (77.5) | 213 (78.9) | 18 (64.3) |
| Supra-marginal | 22 (7.4) | 20 (7.4) | 2 (7.1) |
| Juxta-marginal | 40 (13.4) | 32 (11.9) | 8 (28.6) |
| Missing | 5 (1.7) | 5 (1.8) | 0 (0.0) |
| **Vestibular-Lingual Position** (baseline), N (%) |  |  |  |
| Correct | 249 (83.6) | 225 (83.3) | 24 (85.7) |
| Too vestibular | 22 (7.4) | 18 (6.7) | 4 (14.3) |
| Too lingual | 27 (9.1) | 27 (10.0) | 0 (0.0) |
| Missing | 0 (0.0) | 0 (0.0) | 0 (0.0) |
| **Plaque** (baseline), N (%) |  |  |  |
| 0-5 sites/implant | 276 (92.6) | 256 (94.8) | 20 (71.4) |
| 6 sites/implant | 22 (7.4) | 14 (5.2) | 8 (28.6) |
| Missing | 0 (0.0) | 0 (0.0) | 0 (0.0) |
| **Peri-implant health status** (baseline), N (%) |  |  |  |
| Peri-implant health | 21 (7.1) | 20 (7.4) | 1 (3.6) |
| Peri-implant mucositis | 105 (35.2) | 96 (35.6) | 9 (32.1) |
| Pre-peri-implantitis | 95 (31.9) | 89 (32.9) | 6 (21.4) |
| Peri-implantitis | 77 (25.8) | 65 (24.1) | 12 (42.9) |
| Missing | 0 (0.0) | 0 (0.0) | 0 (0.0) |

*Footnote:*

N, number; SD, standard deviation.
